# A comparative study of Widal test with blood culture in the diagnosis of typhoid fever in febrile patients

**DOI:** 10.1186/1756-0500-7-653

**Published:** 2014-09-17

**Authors:** Gizachew Andualem, Tamrat Abebe, Nigatu Kebede, Solomon Gebre-Selassie, Adane Mihret, Haile Alemayehu

**Affiliations:** Department of Medical Laboratory Sciences, College of Medical Sciences, Haramaya University, Harar, Ethiopia; Department of Microbiology, Immunology and Parasitology, School of Health Sciences, Addis Ababa University, Addis Ababa, Ethiopia; Aklilu Lemma Institute of Pathobiology, Addis Ababa University, Addis Ababa, Ethiopia

**Keywords:** Widal test, Blood culture, Sensitivity, Specificity, Positive predictive value, Negative predictive value

## Abstract

**Background:**

Typhoid fever is a major health problem in developing countries and its diagnosis on clinical ground is difficult. Diagnosis in developing countries including Ethiopia is mostly done by Widal test. However, the value of the test has been debated. Hence, evaluating the result of this test is necessary for correct interpretation of the result. The main aim of this study was to compare the result of Widal test and blood culture in the diagnosis of typhoid fever in febrile patients.

**Methods:**

Blood samples were collected from 270 febrile patients with symptoms clinically similar to typhoid fever and visiting St. Paul’s General Specialized Hospitals from mid December 2010 to March 2011. Blood culture was used to isolate *S.typhi* and *S.paratyphi.* Slide agglutination test and tube agglutination tests were used for the determination of antibody titer. An antibody titer of ≥1:80 for anti TO and ≥1:160 for anti TH were taken as a cut of value to indicate recent infection of typhoid fever.

**Results:**

One hundred and eighty six (68.9%) participants were females and eighty four (31.1%) were males. 7 (2.6%) cases of *S. typhi* and 4 (1.5%) cases of *S. paratyphi* were identified with the total prevalence of typhoid fever 4.1%. The total number of patients who have indicative of recent infection by either of O and H antigens Widal test is 88 (32.6%). The sensitivity, specificity, Positive predictive Value and Negative predictive Value of Widal test were 71.4%, 68.44%, 5.7% and 98.9% respectively.

**Conclusions:**

Widal test has a low sensitivity, specificity and PPV, but it has good NPV which indicates that negative Widal test result have a good indication for the absence of the disease.

## Background

Typhoid fever is a systemic prolonged febrile illness caused by certain Salmonella serotypes including *Salmonella typhi, S. paratyphi A, S. paratyphi B* and *S. paratyphi C*. Human beings are the only reservoir host for typhoid fever, and the disease is transmitted by faecally contaminated water and food in endemic areas especially by carriers handling food. The World Health Organization (WHO) estimates about 21 million cases of typhoid fever with >600,000 deaths annually. The cases are more likely to be seen in India, South and Central America, and Africa i.e. in areas with rapid population growth, increased urbanization, and limited safe water, infrastructure, and health systems [1, 2].

Accurate diagnosis of typhoid fever at an early stage is important not only for diagnosis of etiological agent, but also to identify individuals that may serve as a potential carrier, who may be responsible for acute typhoid fever outbreaks [3]. Options for the diagnosis of typhoid fever are clinical signs and symptoms, serological markers, bacterial culture, antigen detection and DNA amplification [4, 5]. Blood, bone marrow and stool culture are the most reliable diagnostic methods but they are expensive techniques and some bacterial culture facilities are often unavailable [6–8]. In many countries including Ethiopia, the Widal test is the most widely used test in typhoid fever diagnosis because it is relatively cheaper, easy to perform and requires minimal training and equipment [9, 10].

Although Widal test has been in use for more than a century, the value of the test to diagnose typhoid fever has been debated for as many years as it has been available [11]. It relies classically on the demonstration of a rising titer of antibodies in paired samples 10 to 14 days apart. In typhoid fever, however, such a rise is not always demonstrable, even in blood culture-confirmed cases [11]. In addition, Interpreting the test has been such a problem that different cut-offs have been reported from different places [9, 12]. Furthermore, patient management cannot wait for results obtained with a convalescent-phase sample. For practical purposes, a treatment decision must be made on the basis of the results obtained with a single acute-phase sample [7, 13]. So evaluating the result of a single Widal test is necessary for correct interpretation.

This study was carried out to evaluate the value of a single acute-phase Widal test result by blood culture for the diagnosis of typhoid fever in febrile patients in St. Paul’s General Specialized Hospital, Addis Ababa, Ethiopia.

## Methods

### Study area and period

The study was conducted in St. Paul’s general Specialized Hospitals from December 2010 to March 2011. St. Paul’s Hospital, the second largest public hospital in Ethiopia, is located in Addis Ababa. It has 392 beds, with an estimated 800 clinical and nonclinical staffs provide care to more than 110,000 people each year. St. Paul’s Hospital receives referrals from around the country and is under the guidance of the Ethiopian Federal Ministry of Health.

### Study design and patient population

A prospective study on febrile patients was conducted in which patients were screened for typhoid fever and suspected patients were enrolled in the study, then blood sample were collected and tested for confirmation of the disease. Patients were screened by their physician for the clinical symptom of typhoid fever which is fever of 2 or more days before admission accompanied by other clinical symptoms of typhoid fever in the absence of any other known febrile illnesses. Febrile patients whose presumptive clinical diagnosis were typhoid fever and sent to the laboratory by their physician for Widal test were included in the study. However, those febrile patients who had received antibiotic treatment for their symptom within two weeks before coming to the hospital and those who diagnosed for other known febrile illness were not included in this study. By using these inclusion and exclusion criteria about 277 suspected febrile patients were recruited for this study then data and blood sample were collected from these 277 patients.

### Blood sample collection and inoculation

Using a sterile syringe and needle, about 8–10 ml of blood from each adult study subject and about 3–5 ml of blood from each young child was collected. Then 5–7 ml from adults and 2-3 ml of blood from children was dispensed into the culture medium bottle containing 45 ml of Tryptic Soy broth (OXOID, England) mixed with the broth and then incubated at 37°C.

### Sub culturing and biochemical identification

After 24 hours incubation sub-culturing was performed from the Typtic Soya broth on XLD agar (OXOID, England). After overnight incubation positive cultures were proceed further while Negative broth cultures were incubated for seven days and sub cultured before reported negative. Suspected colonies obtained on the above media were screened by biochemical tests using Triple Sugar Iron agar (TSI) (BBL™), citrate utilization test, motility (Difco™), urease test (Himedia ltd. India) and lysine decarboxylation (LDC) [Difco™] test.

### Widal test

Qualitative slide agglutination and semi quantitative tube agglutination (titration) were performed using febrile antigen kits of *Salmonella typhi* (Chromatest Febrile Antigens kits, Linear chemicals, Barcelona, Spain). The slide agglutination test is used as a screening test for the presence of anti TO and anti TH antibodies in the patient’s serum. For the slide agglutination test a drop of *Salmonella typhi* O and H antigens are added on a drop of serum on card and rotated at 100 rpm for one minute and reported as reactive or non reactive. For those slide agglutinations whose results are reactive and weakly reactive titer was determined. In the tube agglutination test (titration), serum sample was serially diluted by using fresh 0.95% saline preparation from 1:20 to 1:640 for anti TO and anti TH separately in 12 test tubes. Then a drop of O antigens and H antigens are added in the test tubes, equal amount in all. Based on the manufacturer manual, an antibody titer of 1:80 and higher for anti TO and 1:160 and higher for anti TH antibodies were taken as a cut of value to indicate recent infection of typhoid fever.

### Quality controls

Standard operational procedures were followed during processing of each sample and all the instruments used for sample processing were checked every morning for proper functioning. *E.coli* ATCC 25922 was used as a reference strain.

### Data analysis

Statistical software package (SPSS Version 16) was used for the analysis of the data. Sensitivity, Specificity, Positive Predictive Value (PPV), and Negative Predictive Value (NPV) were calculated for Widal test.

### Ethical considerations

Ethical clearance was obtained from Research Ethical committee of the department of Microbiology, Immunology and Parasitology of Addis Ababa Unversity. Permission was also obtained from the St.Paul’s General Specialized Hospitals administration. Data and sample were collected after informed consent was obtained from each volunteer and guardian.

## Results

Although 277 febrile patients from the Hospital involved in the study data from 270 patients (68.9% female) were analysed, because three missed due to insufficient serum samples to perform Widal test, other three missed due to incomplete sociodemographic data, and one missed due to both insufficient serum sample and incomplete sociodemographic data. The study participants’ age ranged from 8–80 years (M = 35.82 ± 12.4 [SD]) and most of them were 15–19 years (94.3%).

### Qualitative slide agglutination Widal test

Qualitative slide agglutination Widal test was performed in the hospital laboratory as a primary screening test of serum for presence or absence of the O antigen and H antigens of *S.typhi*. Slide agglutination reaction for O antigen showed that 127 (47.0%) of the patients had reactive agglutination result and 72 (26.7%) have reactive agglutination reaction for H antigen (Table 1).

**Table 1:** **Qualitative slide agglutination reaction results of Widal test of febrile patients suspected of typhoid fever in St. Paul’s hospital**

Reaction result	O antigen		H antigen	
	Frequency	(%)	Frequency	(%)
Reactive	127	(47.0)	72	(26.7)
Weakly reactive	33	(12.5)	55	(20.4)
Non reactive	110	(40.7)	143	(53.0)
**Total**	270	(100.0)	270	(100.0)

One hundred fifty three (53.0%) patients had non reactive reaction result for H antigen of *Salmomella typhi*. Sixty six (24.4%) patients had reactive reaction for both O and H antigens while 61 (22.6%) have reactive only for O antigen. Only 6 (2.2%) of patients have reactive reaction for H antigen only. Overall, 133 (49.3%) patients had reactive slide agglutination test by either or both of O and H antigens.

Among the weakly reactive results, 26 (9.6%) patients had weakly reactive reaction result for both O and H antigen. Six patients with weakly reactive reaction result for O antigen had non reactive reaction result H antigen while one patient with weakly reactive reaction result for O antigen had reactive reaction result H antigen. From 55 patients with weakly reactive reaction result for H antigen, 25 had reactive reaction result for O antigen while only four had non reactive result for O antigen.

### Semiquantitative tube agglutination test (titration)

Titer was performed for those patients whose slide agglutination test result indicated reactive and weakly reactive reactions. One hundred sixty 160 (59.3%) patients had reactive and weakly reactive reaction for anti TO antibody and 127 (47.0%) had reactive and weakly reactive for anti TH antibody. The frequency distribution of titration result is presented on Table 2.

**Table 2:** **The frequency distribution of semi quantitative slide agglutination titration test of Widal test in febrile patients suspected of typhoid fever in St. Paul’s hospital**

Titer	O antigen			H antigen		
	Frequency	% (n = 160)	% from total (n = 270)	Frequency	% (n = 127)	% from total (n = 270)
**No agglutination**	40	25.0	14.8	37	29.1	13.7
**1:20**	32	20.0	11.9	33	26.0	12.2
**1:40**	15	9.4	5.6	5	3.9	1.9
**1:80**	42	26.3	15.6	14	11.0	5.2
**1:160**	21	13.0	7.8	26	20.5	9.6
**1:320**	6	3.8	2.2	12	9.4	4.4
**1:640**	4	2.5	1.5	0	0	0
**Total**	160	100	59.3	127	100	47.0

Serum from 40 (25.0%) patients with reactive (12/40) and weakly reactive (28/40) reaction of slide agglutination for anti TO antibodies did not show any agglutination in tube agglutination titration test. Similarly, 37 (29.1%) of patients with reactive (12/37) and weakly reactive (25/37) reaction for anti TH did not show any agglutination reaction in tube agglutination test. Among those who had agglutination reaction results, 42 (15.6%) had titer of 1:80 for O antigen and 33 (26.0%) had titer of 1:20 of H antigen. There was no titer of 1:640 and higher observed for H antigen but there were only 4 (1.5%) patients whose titer of O antigen was 1:640 and higher.

Antibody titer of 1:80 for O antigen and 1:160 for H antigens were taken as cut of values to indicate recent typhoid infection (positive titer). Taking O ≥ 80 as a cut of value, we found 73 (27%) patients had indicative of recent typhoid infection and taking H ≥ 160 as cut of value, 37 (13.7%) patients had recent typhoid infection. The total number of patients who had indicative of recent infection by either of O and H antigens is 88 (32.6%). Among these 20 (7.4%) patients had antibody titer indicative of recent infection by both O (≥1:80) and H (≥1:160) antigen tests.

The agreement between qualitative slide agglutination and semi quantitative tube agglutination test (titration) indicates that there was a moderate agreement between slide agglutination test and tube agglutination titer for O antigen (Kappa = 0.406) and a fair agreement for H antigen slide agglutination and tube agglutination titer (Kappa = 0.311). In doing these, weakly reactive slide agglutinations reactions were considered as reactive because their titer was determined.

### Blood culture

Of the 270 blood cultures, only seven (2.6%) *S. typhi* were isolated from the patients while *S. paratyphi* were identified from four (1.5%) patients. The blood cultures of fifty one (18.9%) patients’ were positive for bacteria other than salmonella species (Table 3).

**Table 3:** **The distribution of blood culture results of febrile patients suspected of typhoid fever in St. Paul’s hospital**

Bacteria	Number of isolates (%)
***S. typhi***	7 (2.6)
***S. paratyphi***	4 (1.5)
**Non typhoidal salmonella**	7 (2.6)
**Other bacteria**	51 (18.9)
**Negative blood culture**	201 (74.4)
**Total**	270 (100.0)

Based on the above results of Widal test and blood culture for *Salmonella typhi* and *Salmonella paratyphi*, an evaluation of Widal titration results for the diagnosis of typhoid fever was performed for O (≥1:80) and H (≥1:160) antigens tube agglutination test results (Table 4).

**Table 4:** **The distribution of anti TO and anti TH antibody titers among culture positive febrile patients in St. Paul’s hospital**

Widal value	***S. typhi***(n = 7)	***S. paratyphi***(n = 4)	Non typhoidal salmonella species (n = 7)	Other pathogenic bacteria (n = 51)	Negative blood culture (n = 201)
**Positive O titer**	5 (71.4%)	2 (50%)	3 (42.9%)	17 (33.3%)	46 (27.0%)
**Positive H titer**	2 (28.6%)	0 (0%)	2 (28.6%)	9 (17.6%)	25 (12.4%)

Anti TO agglutination titer of 1:80 and higher were detected among 5/7 (71.4%) of culture confirmed typhoid cases by *S.typhi* as compared with 2/4 (50%) of *S. paratyphi* and 3/7 (42.9%) of non typhoidal salmonella. Forty six (27%) patients with a negative blood culture result had a positive Widal titer of anti TO while 25 (12.4%) of them had a positive titer of anti TH. The antibody titer of culture confirmed typhoid fever caused by S. typhi is presented in Table 5. There is no antibody titer of 1:640 and higher observed among culture confirmed cases in both O and H antigens.

**Figure 1:**
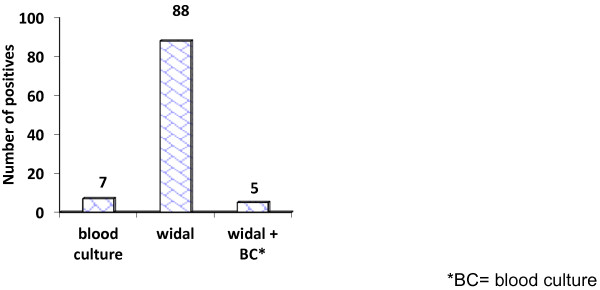
**Diagnostic result of typhoid fever by blood culture and Widal titration of febrile patients suspected of typhoid fever in St. Paul’s hospital, December 2010-March 2011.** *BC= blood culture.

The overall patients which have positive titer for either or both of O and H antigens, and culture confirmed typhoid fever cases were presented on Figure 1.

**Table 5:** **The sensitivity, specificity, PPV, and NPV of titers of anti TO (≥1:80) and anti TH (≥1:160) Widal tests for diagnosis of typhoid fever from febrile patients in St. Paul’s hospital**

Measurement	O antigen (%)	H antigen (%)
Sensitivity	71.4	28.57
Specificity	74.1	86.3
PPV	6.8	5.2
NPV	98.9	97.8

The overall Widal positive titer among culture confirmed cases of *S.typhi* are 5 which all of them have positive titer of anti TO and two of these have also positive titer for anti TH. So the sensitivity, specificity, PPV and NPV of the overall positive titer was 71.4%, 68.44%, 5.7% and 98.9% respectively.

## Discussion

The sensitivity and specificity of Widal titer of anti TO 1:80 and higher in this study were about 71.4% and 74.1% respectively and 28.6% and 86.3% for ant TH titer of 1:160 and higher. The overall sensitivity of titer positive Widal test was about 71.4%, similar with anti TO titer because there was no only anti TH titer positive culture proven typhoid fever identified. This is similar with the study conducted in the endemic area of Vietnam by Olsen *et al*. for the evaluation of serodiagnostic assay of acute enteric fever [14]. Another study done in Kenya has shown that Widal testing done on acute phase serum of patients suspected to has typhoid fever had limited diagnostic capability given its low sensitivity in which among all typhoid cases only 26% had diagnostic titer while 53.6% had O and H titer less than 1:40 [15].

With the cut off value of anti TO ≥1:80 and anti TH ≥ 1:160 Widal titer in this study, Widal test had relatively good NPV (98.9%), but PPV was very low (5.7%). Positive predictive value is more important than other measure of clinical diagnostic methods because it gives the proportion of patients with positive test results that are correctly diagnosed but it is highly affected by a prevalence of the disease. In this study only 7 (2.5%) had culture proven febrile typhoid fever. So a negative Widal test result has a good predictive value for the absence of the disease but a positive result would have a low predictive value for the presence of typhoid fever [9].

A similar study conducted in Egypt indicates that a negative result of Widal test would have a good predictive value of the disease (NPV = 98%) but positive result would have a very low predictive value for typhoid fever (PPV = 5.7%) [16]. Low sensitivity for Widal test may also be related to the data collection time. In the current study Widal test was performed just at the admission of the patient in the hospital.

False positive results of Widal titer were so high in this study (PPV = 5.7%). These false positive results may be associated with cross reacting antibodies from serum of febrile patient other than typhoid fever. In a study conducted in Cameroon to study the prevalence of typhoid fever of febrile patients with clinically compatible symptom of typhoid fever, 45% of the patients the true diagnosis of malaria but only 2.5% of the patients had culture proven typhoid fever [4].

On the other hand, the presence of Widal agglutination under condition of negative malaria smear, negative *S. typhi* culture and without prior immunization against typhoid suggests that other infections may also share common antigenic determinant with *S. typhi* [11]. Typhus, *C. neoformance* meningitis, immunological disorder and chronic liver disease are best example for this [5]. A similar study conducted in Nigeria in apparently healthy freshman students indicates a higher significant titer of antibody for anti TO and anti TH antibodies of *S.typhi* [17]. This may have two negative outcomes in the patient and also in the community. One is that patients are treated (mismanaged) for salmonella having another febrile disease which in turn results in the development of drug resistance [18]. The other is the highly fatal disease of febrile illness such as malaria, non typhoidal salmonellosis, endocarditis and urinary tract infection may be missed [5].

False negative results were also found in this study. Two cases among seven culture confirmed typhoid fever cases had a negative titer. The false negative Widal test results were there probably because blood was collected too early in the disease processes, or inoculated bacterial load is inadequate to induce the antibody production [11]. Previous antibiotic treatment may also contribute to negative Widal agglutination test but there was no patient who explained taking antibiotic within two weeks before coming for the diagnosis during this study.

The positivity of slide agglutination and tube titration in this study was about 49.3% and 38% respectively. Similar positive results were obtained by slide agglutination reaction. Statistically there was moderate agreement (kappa = 0.406) between slide agglutination and tube agglutination titer of anti TO and fair agreement (kappa = 0.311) for anti TH. A study conducted in Jimma, south-western Ethiopia, indicated fair agreement (kappa = 0.225) for anti TO and poor agreement (kappa = 0.06) for anti TH [19]. The current study was conducted in febrile patients while Mamo and his colleagues conducted on healthy population, and this could be one reason for the agreement differences. But still the agreement of slide agglutination and tube titration was very low.

The slide agglutination test is rapid and is used as a screening procedure. An initial positive screening test requires the determination of the strength of antibody. But in many developing countries where the disease is endemic a laboratory professional performs the test, makes diagnosis and reports as positive or negative (reactive and non reactive) [11]. This is also the case of St. Paul’s hospital where this study was conducted. Normally the result of Widal test should be reported as either of ‘agglutination’ or ‘no agglutination’ and if agglutination is present, in titers (1:20, 1:40…) rather than in reactive or non reactive terms. This type of reporting may be misleading and contribute to the incorrect interpretation of the test result by the physicians [11].

In addition to *S.typhi* and *S.paratyphi* other bacteria were identified from blood culture of the febrile patients who had positive or negative Widal titer. In the current study seven (2.6%) cases of non typhoidal salmonella and 51 (18.8%) cases of other bacteria were identified from blood culture. The result of non typhoidal salmonella (2.6%) was similar to a study done in Tanzania; the study identified 2.9% of non typhoidal salmonella from blood culture [9, 20]. Positive Widal titers were also seen in cases of nontyphoidal salmonella and in blood culture positive cases for other bacteria. Three out of 7 (42.9%) of nontyphoidal salmonella cases and 17 of the 51 (33.3%) other bacteria positive cultures had a positive titer of anti TO [21].

## Conclusions

The qualitative slide agglutination tests had a moderate agreement with standard tube agglutination test (titration). Therefore, laboratories should perform the standard laboratory procedure of Widal test and follow the standard reporting instead of in ‘reactive’ and ‘non reactive’ terms. The sensitivity, specificity, PPV and NPV of Widal test were 71.4%, 68.44%, 5.7% and 98.9% respectively. A high antibody titer development is also seen in nontyphoidal febrile infections. In addition Widal test in the labratory should also be performed using O and H antigens of *S.paratyphi A, S.paratyphi B* and *S.paratyphi C.* Nevertheless, using Widal test as the only laboratory test for the diagnosis of typhoid fever will result in misleading diagnosis.
